# Developing Broadband Microstrip Patch Antennas Fed by SIW Feeding Network for Spatially Low Cross-Polarization Situation

**DOI:** 10.3390/s22093268

**Published:** 2022-04-24

**Authors:** Farzad Karami, Halim Boutayeb, Ali Amn-e-Elahi, Alireza Ghayekhloo, Larbi Talbi

**Affiliations:** Département d’Informatique et d’Ingénierie, Université du Québec en Outaouais, Gatineau, QC J8Y 3G5, Canada; halim.boutayeb@uqo.ca (H.B.); a.amn-e-elahi@semnan.ac.ir (A.A.-e.-E.); ghaa10@uqo.ca (A.G.); larbi.talbi@uqo.ca (L.T.)

**Keywords:** antenna array, broadband, cross-polarized fields, satellite application, SIW

## Abstract

A stacked multi-layer substrate integrated waveguide (SIW) microstrip patch antenna with broadband operating bandwidth and low cross-polarization radiation is provided. A complete study on the propagating element bandwidth and cross polarization level is presented to demonstrate the importance of the design. The proposed antenna includes three stacked printed circuit board (PCB) layers, including one layer for the radiating 2 × 2 rectangular patch elements and two SIW PCB layers for the feeding network. There are two common methods for excitation in cavity-backed patch antennas: probe feeding (PF) and aperture coupling (AC). PF can be used to increase the bandwidth of the antenna. Although this method increases the antenna’s bandwidth, it produces a strong cross-polarized field. The AC method can be used to suppress cross-polarized fields in microstrip patch antennas. As microstrip patch antennas are inherently narrowband, the AC method has little effect on their bandwidth. This paper proposes an antenna that is simultaneously fed by AC and PF. As a result of this innovation, the operating bandwidth of the antenna has increased, and cross-polarization has been reduced. Actually, the combination of probe feeding and aperture coupling schemes leads to achieving a broadband operating bandwidth. The arrangement of radiator elements and cavities implements a mirrored excitation technique while maintaining a low cross-polarization level. In both numerical and experimental solutions, a less than −30 dB cross-polarization level has been achieved for all of the main directions. A fractional impedance bandwidth of 29.8% (10.55–14.25 GHz) for $S_{11}$ < −10-dB is measured for the proposed array. Simulated and measured results illustrate good agreement. Having features like low cost, light weight, compactness, broadband, integration capabilities, and low cross-polarization level makes the designed antenna suitable for remote-sensing and satellite applications.

## 1. Introduction

Microstrip patch antennas (MPAs) are broadly used in various types of applications. These antennas have many advantages like low profile, low cost, and easy integration with other devices [1,2,3,4,5,6,7]. However, traditional MPAs usually suffer from some drawbacks like narrow operating bandwidth, low efficiency, and robust cross-polarized fields [8].

The operating bandwidth of single-feed MPAs could be increased by using thick dielectric materials with low dielectric relative permittivity [9], creating resonance slots [10], parasitic patches [11], or advanced electromagnetic structures [12,13,14,15,16]. However, these approaches could increase the surface currents of MPAs, which could have a negative impact on the antenna’s radiation characteristics and efficiency.

Microstrip lines and coplanar waveguides (CPWs) are often used to feed conventional microstrip patch antenna arrays. The operating bandwidth of conventional MPAs with these planar feeding networks is usually less than 5%. By increasing operating frequency, these planar antennas often face many serious problems and challenges. In particular, in higher frequencies, unwanted radiations have suddenly appeared from the feeding network, and the antenna’s efficiency has increasingly dropped [17,18,19,20,21,22].

The radiation efficiency of wireless communication systems is one of the most important parameters that can increase their reliability. The antenna radiation efficiency in satellite and remote-sensing applications is essential due to the inherent losses in long-distance communications. Limiting the antenna’s efficiency can have a direct impact on bandwidth [23,24,25] and gain [26,27].

MPA arrays are commonly fed by microstrip lines. Microstrip lines are typically preferred because they facilitate the routing of low-profile feeding networks. They do, however, introduce unwanted radiation losses, which could have a significant impact on the antenna’s radiation performance and reduce its radiation efficiency.

Substrate integrated waveguide (SIW) technology is one of the best alternatives for microstrip lines and CPWs at higher frequencies. Despite the fact that SIWs require more space than microstrip networks, they provide reliable performance at a higher frequency. In recent years, SIW technology has been used for a wide variety of applications due to its advantages, such as low cost, low loss, and ease of integration. Compared to hollow rectangular waveguide structures, SIW structures have many of the same features and similar radiation characteristics, but they are much less expensive. SIW technology, therefore, could be an effective replacement for RWG. Various types of microwave and millimeter-wave components, such as antennas, feeding networks, filters, etc., have been implemented using SIWs [28,29,30,31,32].

Different strategies have been used to feed MPA with SIW feeding networks. Vertical integration between MPA and an SIW cavity is one of the most effective methods [33,34]. This method leads structures to be compact. These architectures are well known cavity-backed patch antennas. In cavity-backed patch antennas, two different strategies can be used to excite radiating elements; 1. probe feeding (PF) and 2. aperture coupling (AC).

Although, by using the PF method, the impedance characters can be significantly improved, these structures still suffer from high cross-polarization levels [35,36]. Surface currents and higher mode excitation produce robust cross-polarization fields in these antennas. More detailed investigations on conventional MPAs confirm that there are serious concerns about the cross-polarization radiations in these antennas. In rectangular microstrip patch antennas, the cross-polarization level at the H-plane is usually higher than at the E-plane. The cross-polarization level in these antennas and in a range of beamwidth ±25 degrees is usually between −10 and −18 dB.

Exciting patch radiator elements by the PF method leads to generating strong cross-polarized fields on the antenna surface. These strong cross-polarized fields can degrade antenna performance. Moreover, they may increase back lobe radiation in the radiation patterns or even decrease the antenna radiation efficiency [37].

Using an AC method instead of PF can significantly suppress the cross-polarization levels [38]. In comparison with PF, this method does not excite surface currents on the antenna, so it produces radiation patterns with low cross-polarization levels. However, AC has no effect on increasing bandwidth because the operating bandwidth of patch antennas is inherently narrow. In [38], a microstrip patch antenna has been reported with an AC feeding method. Excellent radiation characteristics have been obtained for this antenna. Such structures usually have radiation patterns for which the isolation between their co- and cross-polarization patterns is less than 25 dB. However, their operating bandwidth is usually narrow at less than 5%.

In this paper, a microstrip patch array is presented that uses a combination of PF and AC techniques. Using these two techniques simultaneously increases the impedance bandwidth of the proposed design and suppresses its cross-polarization radiations. In the proposed structure, the radiation elements are surrounded by a small space, which results in a compacted structure and higher efficiency. With 0.5 λ_0_ element spacing, the 2 × 2 proposed array can be easily extended to a large array by using it as a sub-array. Hence, the proposed array could be useful in array applications requiring small element spacing, wide operating bandwidth, and lower cross-polarization levels. This structure has a geometric shape that allows it to be used in a variety of systems, such as monopulse systems, remote sensing, and satellite applications. This paper is organized as follows. Section 2 describes the design procedure of the proposed array. In Section 3, the simulated and measured results of the 2 × 2 proposed array are provided and discussed. Section 3 focuses on antenna characteristics, especially in terms of cross-polarization behavior. Finally, the paper is briefly discussed in Section 4.

## 2. Structure Topology

### 2.1. Subsection

**Figure 1:**
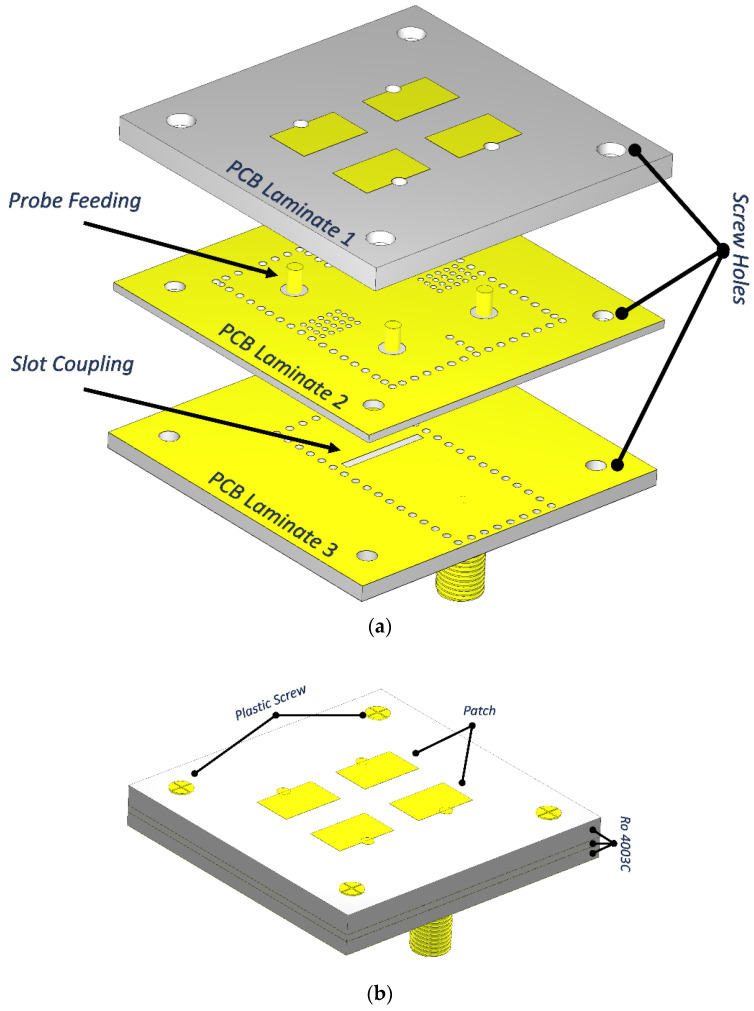

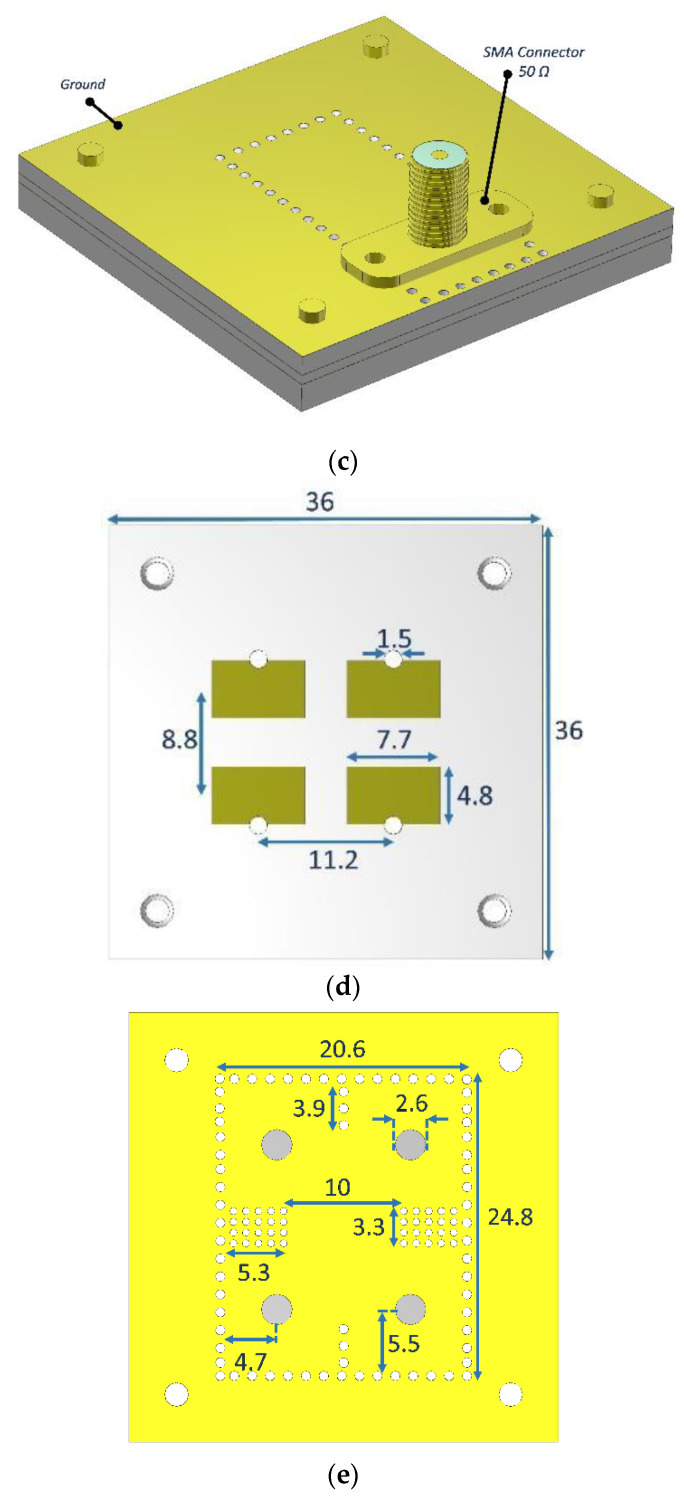

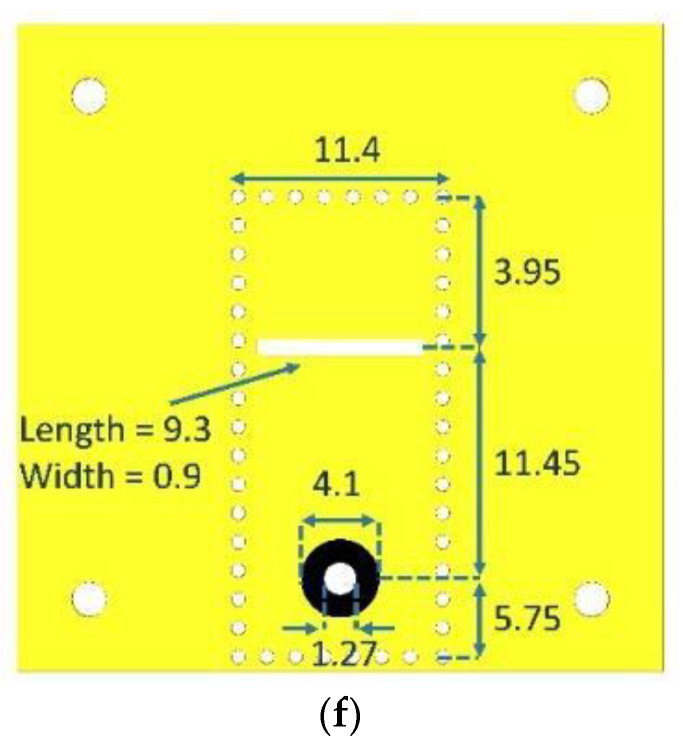
Configuration of the proposed 2 × 2 patch array. (**a**) Exploded view, (**b**) top view, (**c**), back view, and top views of the (**d**) top layer, (**e**) middle layer, and (**f**) bottom layer.

Figure 1a shows the exploded view of the proposed antenna. Other views of the proposed structure are depicted in other parts of the figure. This antenna array includes three stacked PCB layers of Ro4003c dielectric material ($\varepsilon_{r}$ = 3.55 and tanδ = 0.0027) from top to bottom. The first PCB is considered as the antenna layer, while the second and third PCBs are the feeding network. The top view of each layer is shown in Figure 1d–f.

In the proposed design, by using the PF configuration, the microstrip patches are excited. On the common ground between layers 2 and 3, there is a slot, which splits the electromagnetic (EM) power into cavities on the middle layer. In this mechanism, the distance between center to center of adjacent radiating elements in the E-plane is 8.8 mm, i.e., 0.476 λ_0_, whereas distance in the H-plane is 11.2 mm, i.e., 0.36 λ_0_, where λ_0_ is the free space wavelength at 12.5 GHz.

### 2.2. Feed Network Topology

A square topology is used for the EM power distribution network based on four SIW cavities. SIW cavities are formed by inductive windows. This feeding network is designed by considering several primary dimensions as follows. To control the leakage of the EM wave, the diameters of cylindrical metalized holes (vias) are selected by these conditions: S/d ≤ 2, and d/$\;\lambda_{0}\;\leq \;0.1$, where $\lambda_{0}$ is the free-space wavelength at the center frequency. The resonance frequency is determined by the cavities’ dimensions.

The resonance frequency is determined by the cavity dimensions. The following relationships could be used to calculate the SIW cavity’s initial dimensions: [39]

(1) $$f_{mnp}\;\frac{C}{2\sqrt{\varepsilon_{r}}}\sqrt{{\left(\frac{m}{Wcavity_{eff}}\right)}^{2}+{\left(\frac{n}{Lcavity_{eff}}\right)}^{2}+{\left(\frac{p}{h}\right)}^{2}}$$

(2) $$Wcavity_{eff}=W_{Cavity}-1.08\frac{d_{1}{}^{2}}{P_{1}}\;+0.1\frac{d_{1}{}^{2}}{W_{Cavity}}$$

(3) $$Lcavity_{eff}=L_{Cavity}-1.08\frac{d_{1}{}^{2}}{P_{1}}\;+0.1\frac{d_{1}{}^{2}}{L_{Cavity}}$$

The resonant frequency of the proposed cavities is determined by *f_mnp_.* The designed cavities operate when *m = n =* 1 and *p =* 0.

Cavity dimensions are represented by the parameters $W_{cavity}$, $L_{cavity}$ and h in the mentioned formula. Moreover, in free space, C is the speed of light, and *ε_r_* (=3.55) is the dielectric relative permittivity. The resonant mode for proposed cavities is TE_110_.

To minimize the leakage in the SIW layers, vias are used with diameter d = 0.8 mm, and the center-to-center spacing between adjacent vias is S = 1.6 mm.

After primary calculations, CST Microwave Studio was used to optimize the geometries of the proposed cavities in Figure 1 to obtain the desired values and best performance.

**Figure 2:**
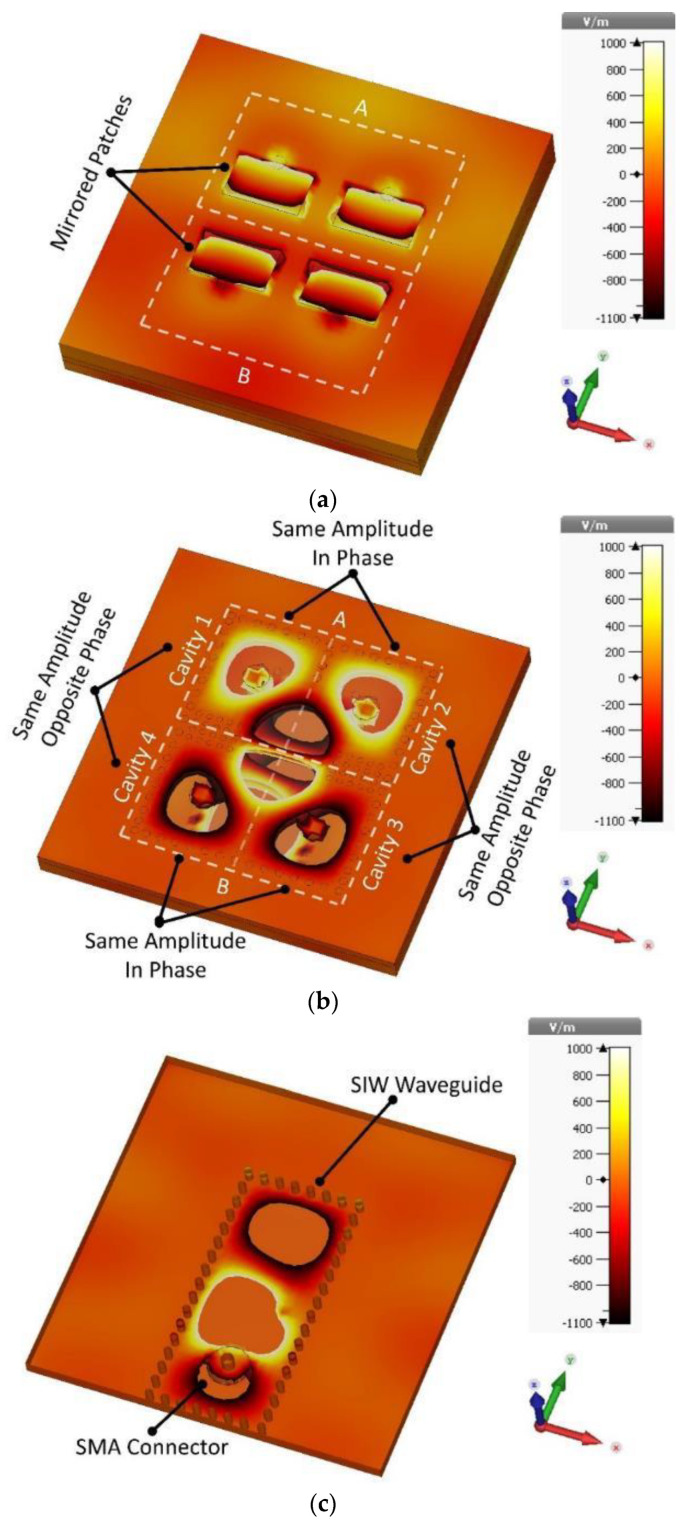
Simulated electric field distributions of the proposed array. (**a**) Top layer, (**b**) middle layer, and (**c**) bottom layer.

As shown in Figure 2, cavities are named 1, 2, 3, and 4. The specified area A contains cavities 1 and 2, while area B contains cavities 3 and 4. The power distribution in the feeding network is as follows. First, EM power flows to the SIW channel through an SMA connector 50 Ω. Then, it enters the cavities through an embedded slot. Cavities 1 and 2 are excited in phase (also cavities 3 and 4 are excited in phase), but there is a 180-degree phase difference between cavities in specified areas A and B.

Figure 2 shows the distribution of EM fields in the proposed structure considering the phase situation. The specified areas A and B have co-polarization radiation without phase difference, while the cross-polarization radiations have a 180-degree phase difference. Arrangement of the patches and the way the distribution of the EM fields in the feeding network contributes to phase cancelation leads to suppressing cross-polarization fields.

## 3. Discussion of Experimental Result

### 3.1. Array Fabrication

**Figure 3:**
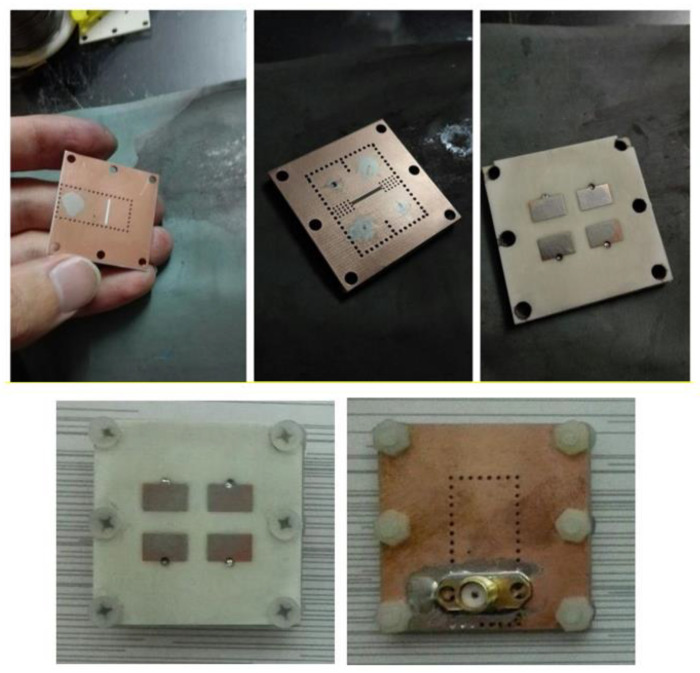
Photographs of the proposed fabricated array.

**Figure 4:**
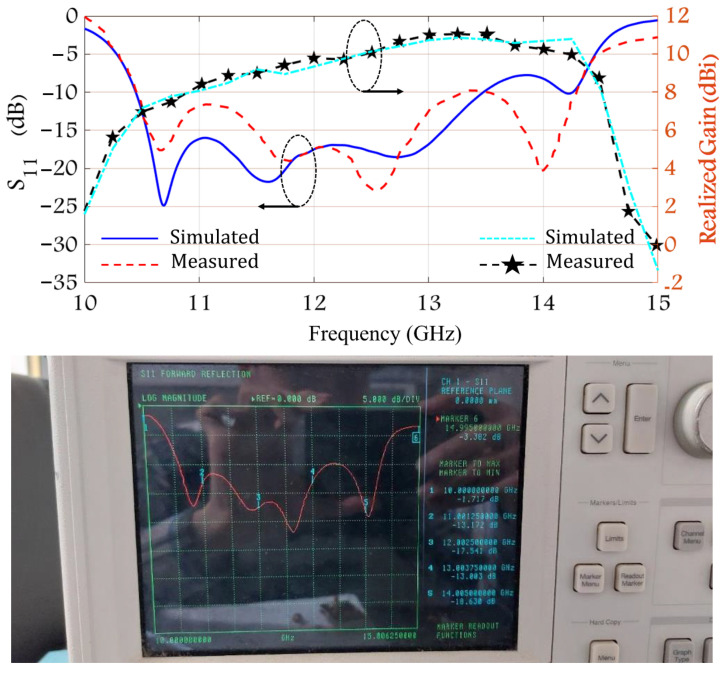
Simulated and measured S_11_ and the realized gain of the fabricated patch array.

To validate the simulated performance of the proposed array, a prototype shown in Figure 3 was fabricated and experimentally characterized. Figure 3 shows the photographs of the fabrication process and fabricated structure. The proposed structure was fabricated in a precise manufacturing process along with the accurate installation of the stacked PCB layers. Figure 4 shows the simulated and measured $S_{11}$ and the realized gain of the proposed array. The measured $S_{11}$ for <−10 dB is from 10.55 GHz to 14.25 GHz, which corresponds to 29.8%. The maximum gain measured for the proposed array is 11 dB at 13 GHz.

### 3.2. Radiation Characteristic of the Proposed Structure

The appearance of robust cross-polarized fields in the radiation characters of patch antennas is always a fundamental challenge for antenna designers. In the conventional patch antennas, excitation of higher modes with orthogonal polarization causes cross-polarized fields in the H-plane. A cross-polarized field in the H-plane is stronger than in the E-plane. Studies show that in the H-plane of patch antennas within the ±25° beamwidth, the isolation between the co- and cross-polarization radiation is around 10–20 dB [37,39].

The EM radiation patterns of the proposed MPA are discussed in this section. The work is mainly focused on the study of cross-polarized fields. When investigating the cross-polarization behavior in the antennas, an important point must be considered. Only checking the isolation between co- and cross-polarization patterns in the two main cutting planes of E- and H- is not sufficient to confirm that an antenna has a low cross-polarization level. In antenna characteristics, the cross-polarization level could be extremely low in both the E- and H-planes, while on the other cutting planes, the cross-polarization level could be extremely high. This behavior can exist in various types of antennas. The distribution of the electric fields on the horn antenna’s aperture clearly indicates that on the E- and H-cutting plane, the cross-polarization level is minimal, while on the other orthogonal cutting planes, strong cross-polarization radiation is observed [40]. More rigorous studies on cross-polarization in microstrip patch antennas indicate that the isolation between co- and cross-polarizations in the E-plane is at its minimum, while its maximum occurs on cutting plane 𝜙 = 70°, somewhere between the E- and H-planes [41,42,43]. Therefore, investigating cross-polarization fields only on the E- and H-cutting planes can cause misjudgments concerning cross-polarization behavior on antennas.

**Figure 5:**
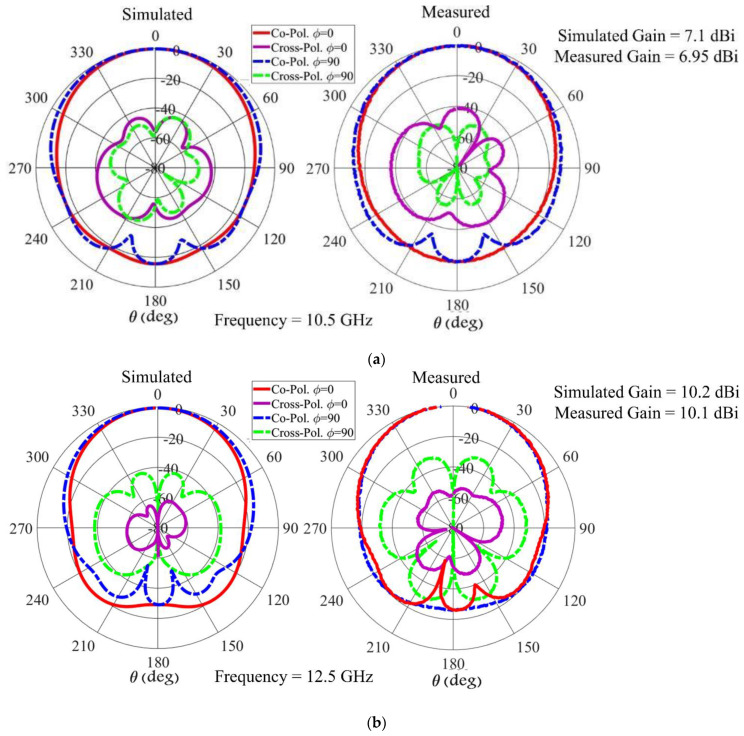

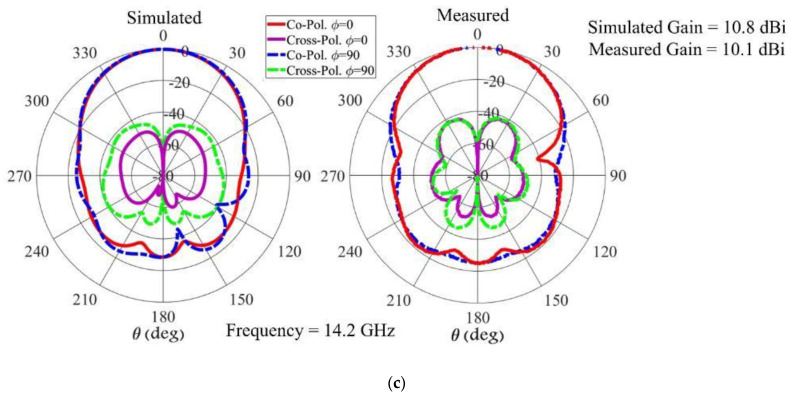

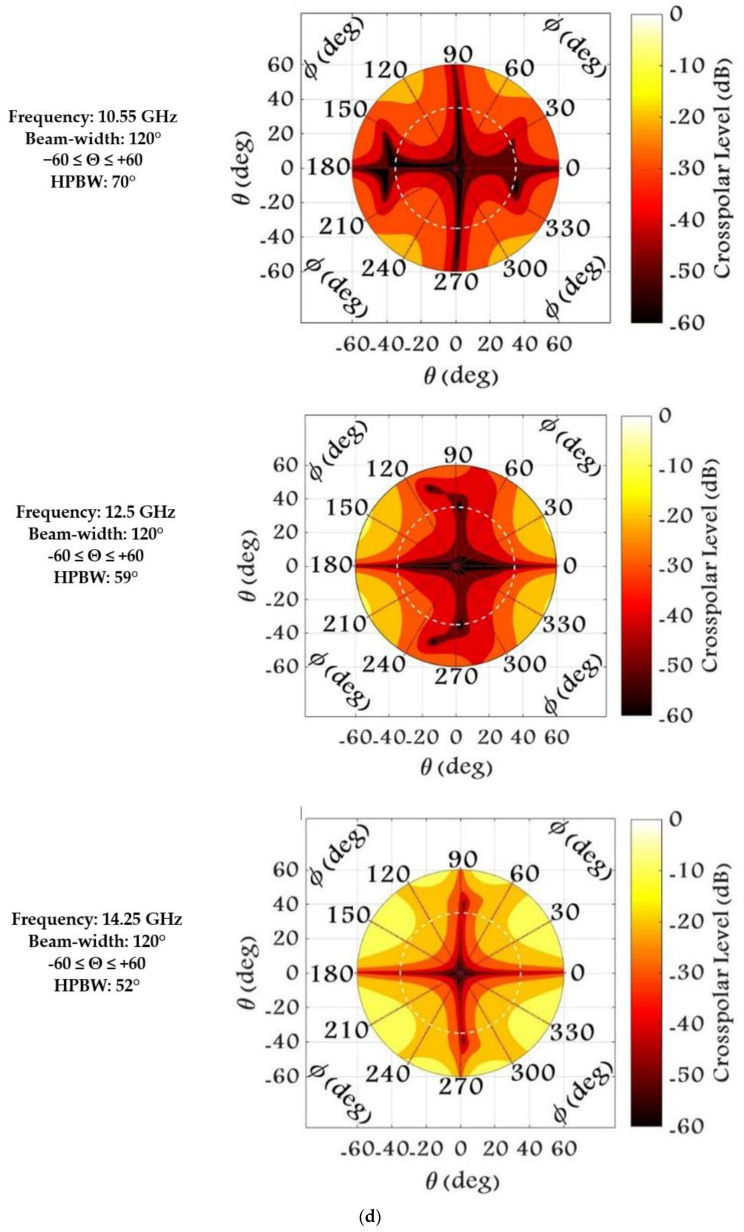
Simulated and measured radiation pattern of the proposed array for different frequencies of (**a**) 10.55, (**b**) 12.5, and (**c**) 14.25 GHz. (**d**) Simulated cross-polarization level of the proposed array versus Ɵ and 𝜙 angles.

In this paper, in order to accurately investigate cross-polarization behavior, as well as ensure its low level on all the planes, contour plots are presented. These contour plots indicate the cross-polarization level on different cutting planes. Then, the simulated and measured radiation patterns of the proposed antenna on the cutting planes 𝜙 = 0° (E-plane) and 𝜙 = 90° (H-plane) are presented. Figure 5d displays the simulated cross-polarization levels of the proposed array versus Ɵ and 𝜙 angles at 10.55, 12.5, 14.25 GHz. These charts are drawn for a 120° (−60° ≤ θ ≤ 60°) beamwidth. In the contour plots, the area specified with a dashed line indicates half-power beamwidth (HPBW). The contour plots confirm that cross-polarization levels at HPBW in the whole operating bandwidth and in all 𝜙 cutting planes are less than 30 dB. Figure 5 also shows the simulated and measured normalized radiation patterns of the proposed structure at 10.55, 12.5, and 14.25 GHz.

### 3.3. Antenna’s Efficiency

In reliable wireless communication systems, the antenna’s efficiency is a significant feature. The impedance bandwidth and gain could be directly limited by this feature. Several single-feed MPA designs have already been proposed. Microstrip lines fed the majority of them. The designs based on microstrip lines present a feasible structure in which the feeding network production is easier while maintaining a low profile. However, as the antenna’s operating frequency rises, the amount of unwanted energy transmission from these feeding lines rises dramatically. This unwanted energy transmission has a significant negative impact on the radiation pattern, resulting in a reduction in radiation efficiency.

Antenna radiation efficiency is described by IEEE standards as “the ratio of the total power radiated by an antenna to the net power accepted by the antenna from the connected transmitter” [44]. Dielectric losses, conductor losses, and unwanted radiation from the feeding network structure are some important aspects that contribute to the reduction of radiation efficiency in a PCB-based propagating element. The effect of the dielectric losses can be diminished by the use of new low-loss tangent dielectrics [45]. This strategy can improve the antenna’s radiation efficiency to some extent, but if the antenna’s feeding network has unwanted radiation losses, the antenna’s radiation efficiency will be significantly reduced. This unwanted energy transmission may have negative effects on the antenna’s far-field pattern along with a growth in cross-polarization level, in addition to lowering antenna efficiency.

The radiation efficiency of large arrays is primarily affected by unwanted radiation and losses from the combinatory feeding structure (such as in parallel feeding topology). It is possible to achieve higher radiation efficiency when minimizing the length of the feeding networks, such as those in series feeding [30,46]. It does, however, usually have a narrow frequency bandwidth, and it can distract the main beam propagation features. The proposed array’s maximum simulated radiation efficiency is 93%. The proposed design has an aperture dimension of 36 × 36 mm^2^, and it has a maximum realized gain of 11 dBi at 13.5 GHz frequency.

In terms of frequency bandwidth, the proposed formation presents wideband radiation elements like the magneto-electric dipole [47], L-probe-fed patch array [48], U-shaped parasitic patches [49], and slot antenna [50]. Although using low temperature co-fired ceramic (LTCC) substrates to implement multi-layer broadband antennas is a viable option, the high cost of this technology keeps it from being widely adopted [51,52].

A single-element X-band patch antenna is presented in [53] and fed by the SIW through a slot on its top wall. This structure has an impedance bandwidth of 8.8%. This single element antenna has a potential use in array applications. It appears that two approaches are available for designing a larger array from a single element in [53]: (1) using a series formation of the feeding structure, such as the one described in [38]. (2) Making use of a corporate feeding network like [54]. According to studies, a broad impedance bandwidth cannot be attained when a series feeding structure is used to design a larger array formation. Reference [38] shows a 6 × 8 patch array fed by an SIW standing wave series feeding structure. The structure describes a proper radiation routine, but it only has a 1.8% operating bandwidth. A 2 × 2 array of microstrip patch antennas is described in [54], which is fed by an SIW corporate feeding network. In fact, four single elements (like the formation presented in [53]) were used to create this array. These elements are fed using three power dividers in an SIW’s corporate feeding network. The array formation cannot be lengthy along x and y due to the large feeding network, so the gains of these arrays are lower than 11.1 dBi. Furthermore, a large area is located around the propagating elements, increasing the structure’s dimension. The radiation efficiency can be severely harmed by the surrounding boundary around the propagating element [55,56,57].

### 3.4. State of the Art Aspects

A comparison between various characteristics of the proposed design and previously reported results in terms of the number of layers, antenna size, operation frequency, impedance bandwidth, and cross-polarization level are provided in Table 1. Differential feeding methods have been studied in literature to suppress cross-polarized fields in MPAs [34,58]. Although the use of these methods effectively reduces the cross-polarization radiation, the bandwidth of these structures is still narrow. In addition, the implantation of some of these methods needs an additional number of excitation ports, which leads to an increase in the measurement costs.

In [36], a 4 × 4 microstrip patch antenna array is reported using the PF method to excite radiating elements. A PF geometry consists of a metal pin and a surrounding ring slot. By changing the diameters of the ring slot and metallic pin, the reactance and conductance can be controlled to achieve a broader impedance matching. Applying the PF method has resulted in a wide operating bandwidth of about 19% for this structure. However, this structure suffers greatly from high cross-polarization levels. So, this structure has a serious limitation to use in some practical applications. In addition, this multi-layer structure has a number of pin couplings and blind screws for internal connection between layers. The fabrication process of this structure is complex, and its assembly requires high precision.

In [39], the AP method is used to excite the patch elements. Applying the AP method causes a uniform distribution of EM fields on the radiation elements, and consequently, the level of cross-polarization is much lower than that of the PF method. Nevertheless, since MPAs are inherently narrowband, the AC method does not have a significant effect on increasing the impedance bandwidth.

In [43], a two-layer power distribution network is utilized to suppress cross-polarization fields in a 4 × 4 patch array antenna. The feeding network of this array operates by changing the propagation mode from TE_20_ to TE_10_. The impedance bandwidth of this five-layer antenna is 12.9%. The used feeding network in this structure is complex. Moreover, the number of PCB layers will be increased by developing this structure to a large array.

There are advantages of the proposed structure can overcome many of the challenges associated with previous designs. This manuscript presents a 2 × 2 array of a broadband MPA with low cross-polarization levels. The presented structure has several features. First, a combination of PF and AC methods has been used to feed this structure. PF allows us to control impedance characters and achieve a higher impedance matching. The experimental results indicate that the operating bandwidth of this MPA array is 29.8%. On the other hand, the aperture coupling method has helped us to provide a very effective mechanism for suppressing cross-polarization without the need to design complex power distribution networks. The reduced cross-polarization is studied for a 3-D half-power beamwidth space. The measured results show that the cross-polarization level for the presented MPA is less than 30 dB in the whole operating impedance bandwidth. The designed array can be easily used as a subarray to design larger arrays. This does not need to increase the number of PCB layers to design larger arrays. The proposed array does not require the use of blind screws or even internal connections and its fabrication process is simple.

## 4. Conclusions

A successful combination of aperture coupling and probe feeding methods is demonstrated in this paper. The main advantages of this compact package are its broadband character and low cross-polarization levels. A mirrored geometry between radiating elements and the feeding network is used to create the 2 × 2 array. This design occupies less space around the radiation elements, which reduces the size of the antenna and improves its efficiency. In this study, the suppressed cross polarization is verified for a 3-D half power beamwidth area in the whole operating bandwidth (10.55–14.25 GHz). For all of the main directions, the cross-polarization level is less than −30 dB. With a 0.5 λ_0_ element spacing, the proposed 2 × 2 array can be easily used as a sub-array to design a large array. Hence, the proposed array could be useful in applications requiring small element spacing, wide operating bandwidth, and lower cross-polarization levels. It has good potential to use the proposed structure as a subarray in a larger array for a wide spectrum of practical applications such as satellite remote-sensing antennas.

## Figures and Tables

**Table 1:** Comparison between previously reported antennas and the proposed antenna.

Ref.	Number of Layers	Number of Ports	Aperture Size ($\lambda^{3}$)	Max Gain (dB)	Frequency (GHz)	Impedance Bandwidth (%)	X-Pol. within HPBW (dB)
[2]	7	1	12.16 × 13.6 × 0.82	30.1	57.5–67	15.3	−50
[4]	2	1	8.16 × 2.28 × 0.13	23.2	14.5–17.4	18.2	−30
[21]	3	1	N. A.	19	23.2–24.8	6.67	−40
[34]	3	4	N. A.	12.89	9.77–10.62	8.3	~−25
[36]	4	1	2.49 × 2.29 × 0.24	17.1	11.2–13.6	19.36	−17
[39]	3	1	1.36 × 1.36 × 0.32	11.1	9.35–10.25	9.18	−40
[43]	4	1	2.25 × 2.25 × 0.48	16.3	9.4–10.7	12.9	−40
[51]	4	1	2.95 × 3 × 0.37	18.6	56.3–66.3	11.7	~−25
[58]	2	1	1.73 × 1.73 × 0.1	12.3	12.2–13.8	12.8	~−17
This Work	3	1	1.48 × 1.48 × 0.16	11	10.55–14.25	29.8	−30

## Data Availability

The data presented in this study are openly available.
